# Correcting and interpreting the effect of cognitive therapy versus exposure in anxiety disorders

**DOI:** 10.1186/1471-244X-12-202

**Published:** 2012-11-20

**Authors:** Shanil Ebrahim, Sheena Bance

**Affiliations:** 1 Department of Clinical Epidemiology and Biostatistics, McMaster University, 1200 Main Street West, Hamilton, Ontario, Canada; 2 Department of Adult Education and Counselling Psychology, University of Toronto, Toronto, Canada

**Keywords:** Cognitive therapy, Exposure, Anxiety disorders, Systematic review, Meta-analysis, Correspondence, Correction

## Abstract

Dr. Ougrin’s evaluation of cognitive therapy versus exposure in anxiety disorders reported a standardised mean difference [SMD] (95% confidence interval [CI]) of 0.52 (0.37, 0.74) for short-term outcomes and 0.46 (0.29, 0.73) for long-term outcomes in social phobia, and 0.88 (0.69, 1.11) for short-term outcomes and 1.05 (0.80, 1.37) for long-term outcomes in posttraumatic stress disorder (PTSD). These were incorrectly meta-analysed. Upon re-analysis, we found that the correct SMD (95% CI) was −0.66 (−1.19, -0.14) for short-term outcomes and mean difference (95% CI) of −29.66 (−46.13, -13.19) on the Social Phobia subscale from the Social Phobia Anxiety Inventory for long-term outcomes in Social Phobia. For PTSD, the SMD (95% CI) for short-term outcomes was −0.13 (−0.36, 0.11) and 0.05 (−0.22, 0.32) for long-term outcomes. However, correcting the errors did not change the interpretation of the findings considerably.

## 

We read with great interest the systematic review evaluating the efficacy of exposure versus cognitive therapy (CT) in anxiety disorders, performed by Dr. Dennis Ougrin [1]. The author meta-analysed 20 randomised controlled trials (RCTs) comparing CT versus exposure in four anxiety disorders and concluded the following: “there appears to be no evidence of differential efficacy between cognitive therapy and exposure in PD [panic disorder], PTSD [posttraumatic stress disorder] and OCD [obsessive compulsive disorder] and strong evidence of superior efficacy of cognitive therapy in social phobia” [1]. 

Upon reviewing the short-term and long-term results of CT versus exposure in patients with Social Phobia, we found two errors. First, the summary effect (standard error [SE]) for the short-term outcome in Hofmann (2004) was incorrectly inputted as −0.28 (0.26). We contacted Dr. Stefan Hofmann [2], obtained the raw data from the published trial, and found that the correct summary effect (SE) was −0.19 (0.27). Second, Dr. Ougrin states, “the overall effect (the end-of-treatment standardised mean difference (SMD), Hedge’s g) is summarised in Figure 7” [1]. The forest plot, however, does not indicate what summary effect was reported in the pooled analysis and thus the assumption was made that it was the SMD. Upon replicating the meta-analysis, we found that the summary effect was incorrectly pooled as an odds ratio (OR), i.e. OR (95% confidence interval [CI]) of 0.52 (0.37, 0.74) for short-term outcomes. Thus, we inputted the correct summary effect for Hofmann 2004 and re-analysed the meta-analysis using SMD for our summary effect, as originally intended. We found that the corrected SMD (95% CI) was −0.66 (−1.19, -0.14) for short-term outcomes. We used the random effects model given that the i^2^ was 56% (Figure 1). Using the Cohen’s d criteria of 0.2 to represent a small effect, 0.5 a medium effect and 0.8 a large effect [3], we found that CT has a medium effect in improving social phobia versus exposure with the lower bounds of the 95% CI in the range of a modest effect, for short-term outcomes.

**Figure 1:**
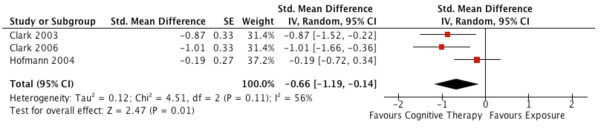
Corrected meta-analysis of Figure 7 in Ougrin (2011): the short-term efficacy of cognitive therapy versus exposure in Social Phobia.

Long-term outcomes for social phobia were also incorrectly pooled as an OR. We found that the corrected SMD (95% CI) was 0.46 (0.29, 0.73) for long-term outcomes. However, the SMD should only be used when pooling different assessments for the same outcome. If the same assessment is reported across trials, the mean difference (MD) is always preferred. Both Clark (2006) and Hofmann (2004) [2,4] reported social phobia subscale scores from the Social Phobia and Anxiety Inventory (SPAI) [5]. Thus, we calculated the MD (95% CI) as −29.66 (−46.13, -13.19) (Figure 2).

**Figure 2:**
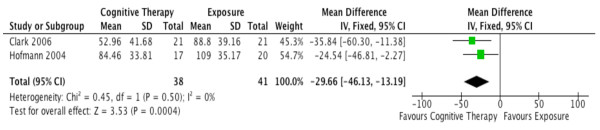
Corrected meta-analysis of Figure 8 in Ougrin (2011): the long-term efficacy of cognitive therapy versus exposure in Social Phobia.

## Additional errors identified by Professor Julio Sánchez-Meca

**Figure 3:**
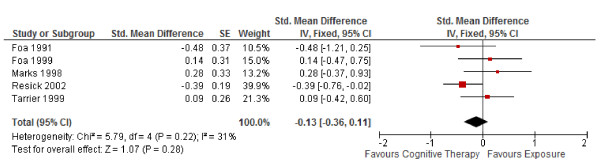
**Corrected meta-analysis of Figure**3**in Ougrin (2011) “Cognitive therapy versus exposure for PTSD.** Meta-analysis: short-term outcomes”.

**Figure 4:**
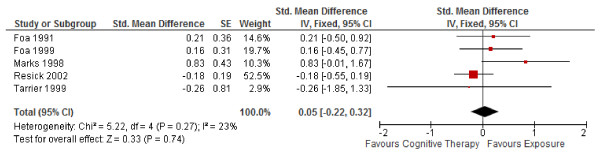
**Corrected meta-analysis of Figure**4**in Ougrin (2011) “Cognitive therapy versus exposure for PTSD.** Meta-analysis: long-term outcomes”.

Professor Julio Sánchez-Meca, who was a reviewer of our article, identified additional errors in Dr. Ougrin’s paper as follows:

1. In Figure 3 in Ougrin (2011), the forest plot was incorrectly pooled as odds ratios when the effect sizes were in fact SMDs (see Table 2 in Ougrin). We found that the corrected SMD (95% CI) was −0.13 (−0.36, 0.11) for short-term outcomes in PTSD (Figure 3).

2. Figure 4 in Ougrin (2011) presents the same error as above. We found that the corrected SMD (95% CI) was 0.05 (−0.22, 0.32) for long-term outcomes in PTSD (Figure 4).

3. On page 4 in Ougrin (2011), second column, fifth paragraph, reporting the results of the meta-analysis for short-term outcomes in panic disorder, an I^2^ of 68% is reported. However, in Figure 5 in Ougrin (2011), an I^2^ of 62% is reported.

4. On page 4 in Ougrin (2011), second column, last paragraph, reporting the results of the meta-analysis for long-term outcomes in panic disorder, an I^2^ of 24% is reported. However, in Figure 6 in Ougrin (2011), an I^2^ of 69% is reported. In addition, the author reported that a fixed effects model was applied when actually, a random-effects model was employed.

## Conclusions

In summary, after correcting and re-analysing the meta-analyses, we found a medium effect in CT improving social phobia in comparison to exposure. It is important to note, however, that correcting the errors did not change the interpretation of the findings considerably in Dr. Ougrin’s paper. Our paper stresses the importance of critically checking one’s data and analyses to ensure validity of results.

## Response

**By** Dennis Ougrin dennis.ougrin@kcl.ac.uk

I would like to thank Ebrahim and Bance for confirming and essentially independently replicating the main results of the meta analysis evaluating the efficacy of exposure versus cognitive therapy (CT) in anxiety disorders [5]. Ebrahim and Bance reviewed the short-term and long-term results of CT versus exposure in patients with social phobia and found a slightly different standardised mean difference (SMD) and standard error [SE] for the short-term outcomes in Hofmann (2004) [1]: -0.19 (0.27) rather then −0.28 (0.26). Ebrahim and Bance did not state how their calculation was made. Since different formulae exist for this calculation it might be helpful if the authors provide the entirety of their calculations rather then only providing the final figure. The authors further used their calculation of the SMD in Hofmann (2004) [1] to re-calculate the pooled SMD, using random effects model instead of the original fixed effects model (due to the increased heterogeneity) and found the pooled SMD to be −0.66 (−1.19, -0.14), slightly greater then the original estimate but with wider confidence intervals. The recalculation of long-term outcomes for social phobia yielded essentially similar results. However, the authors substituted the SMD with the mean difference (MD) from the Social Phobia and Anxiety Inventory (SPAI) as the SPAI results were reported by all three studies used to calculate the pooled SMD [1-6]. This may be a preferred strategy in most cases, however for the purpose of the original meta analysis and a priori decision was made to use the main outcome measure in each study. In both Clark (2003) and Clark (2006) the main outcome measure was a social anxiety composite, hence the decision was to use the SMD instead of the MD. Ebrahim and Bance’s recalculation of the SMD in the PTSD [posttraumatic stress disorder] studies yielded essentially similar results as those in the original meta analysis. I would like to thank the authors for spotting several typos and some reporting inconsistencies which sadly even a very thorough peer review may miss. One important lesson is to check all default settings in the software used for meta analyses. Finally I would like to emphasise that confirmations and independent replications are extremely important and still quite rare in the field of psychological therapies. Both clinicians and researchers may feel more confident that extant literature does indeed indicate no overall difference between cognitive therapy (including behavioural experiments) and exposure for in PD [panic disorder], PTSD and OCD [obsessive compulsive disorder] and points to superior efficacy of cognitive therapy in social phobia.

## Competing interests

The authors declare that they have no competing interests.

## Authors’ contributions

SE and SB prepared, critically revised and approved the final manuscript to be published.

## Authors’ information

SE is a doctoral candidate in the Department of Clinical Epidemiology and Biostatistics at McMaster University in Hamilton, Ontario, Canada. SB is a master’s student in the Department of Adult Education and Counselling Psychology at the University of Toronto in Toronto, Ontario, Canada.

## Pre-publication history

The pre-publication history for this paper can be accessed here:

http://www.biomedcentral.com/1471-244X/12/202/prepub
